# The respiratory enzyme complex Rnf is vital for metabolic adaptation and virulence in *Fusobacterium nucleatum*

**DOI:** 10.1128/mbio.01751-23

**Published:** 2023-12-07

**Authors:** Timmie A. Britton, Chenggang Wu, Yi-Wei Chen, Dana Franklin, Yimin Chen, Martha I. Camacho, Truc T. Luong, Asis Das, Hung Ton-That

**Affiliations:** 1 Molecular Biology Institute, University of California, Los Angeles, California, USA; 2 Department of Microbiology & Molecular Genetics, University of Texas McGovern Medical School, Houston, Texas, USA; 3 Division of Oral & Systemic Health Sciences, School of Dentistry, University of California, Los Angeles, California, USA; 4 Department of Medicine, Neag Comprehensive Cancer Center, University of Connecticut Health Center, Farmington, Connecticut, USA; 5 Department of Microbiology, Immunology & Molecular Genetics, University of California, Los Angeles, California, USA; The University of Kansas Medical Center, Kansas City, Kansas, USA; Medical University of South Carolina, Charleston, South Carolina, USA

**Keywords:** *Fusobacterium nucleatum*, Rnf complex, metabolism, hydrogen sulfide, coaggregation, preterm birth, virulence

## Abstract

The Gram-negative oral pathobiont *Fusobacterium nucleatum* can traverse to extra-oral sites such as placenta and colon, promoting adverse pregnancy outcomes and colorectal cancer, respectively. How this anaerobe sustains many metabolically changing environments enabling its virulence potential remains unclear. Informed by our genome-wide transposon mutagenesis, we report here that the highly conserved *Rhodobacter* nitrogen-fixation (Rnf) complex, encoded by the *rnfCDGEAB* gene cluster, is key to fusobacterial metabolic adaptation and virulence. Genetic disruption of the Rnf complex via non-polar, in-frame deletion of *rnfC* (Δ*rnfC*) abrogates polymicrobial interaction (or coaggregation) associated with adhesin RadD and biofilm formation. The defect in coaggregation is not due to reduced cell surface of RadD, but rather an increased level of extracellular lysine, which binds RadD and inhibits coaggregation. Indeed, removal of extracellular lysine via washing Δ*rnfC* cells restores coaggregation, while addition of lysine inhibits this process. These phenotypes mirror that of a mutant (Δ*kamA*) that fails to metabolize extracellular lysine. Strikingly, the Δ*rnfC* mutant is defective in ATP production, cell growth, cell morphology, and expression of the enzyme MegL that produces hydrogen sulfide (H_2_S) from cysteine. Targeted metabolic profiling demonstrated that catabolism of many amino acids, including histidine and lysine, is altered in Δ*rnfC* cells, thereby reducing production of ATP and metabolites including H_2_S and butyrate. Most importantly, we show that the Δ*rnfC* mutant is severely attenuated in a mouse model of preterm birth. The indispensable function of Rnf complex in fusobacterial pathogenesis via modulation of bacterial metabolism makes it an attractive target for developing therapeutic intervention.

**IMPORTANCE:**

This paper illuminates the significant question of how the oral commensal *Fusobacterium nucleatum* adapts to the metabolically changing environments of several extra-oral sites such as placenta and colon to promote various diseases as an opportunistic pathogen. We demonstrate here that the highly conserved *Rhodobacter* nitrogen-fixation complex, commonly known as Rnf complex, is key to fusobacterial metabolic adaptation and virulence. Genetic disruption of this Rnf complex causes global defects in polymicrobial interaction, biofilm formation, cell growth and morphology, hydrogen sulfide production, and ATP synthesis. Targeted metabolomic profiling demonstrates that the loss of this respiratory enzyme significantly diminishes catabolism of numerous amino acids, which negatively impacts fusobacterial virulence as tested in a preterm birth model in mice.

## INTRODUCTION

A prominent member of the human oral microbiota that is known to harbor over 700 bacterial and fungal species (1, 2), the Gram-negative anaerobe *Fusobacterium nucleatum* plays an integral role in oral biofilm development and dental plaque formation, by virtue of its inherent capacity to adhere to diverse microbial species, notably the *Actinomyces* spp., *Streptococcus* spp., *Porphyromonas gingivalis*, and *Candida albicans* (3–12). Beyond commensalism, *F. nucleatum* is also an opportunistic pathogen that can induce preterm birth, promote tumor growth and metastatic progression of breast cancer cells, proliferation and migration of pancreatic cancer cells, and colorectal cancer (CRC), and is noted for its ability to spread from the oral cavity and colonize many extra-oral sites, including placental, breast, pancreatic, and colorectal tissues (13–17). To date, a number of adhesins have been shown to mediate host-pathogen interactions. These include Fap2 and FadA, with the former aiding fusobacterial binding to tumor- and placenta-expressed Gal-GalNAc (18, 19), while the latter promoting placental colonization and CRC progression (17, 20–22). The other known adhesin RadD is a major fusobacterial virulence factor that not only mediates polymicrobial interaction (or coaggregation), a process that is inhibited by arginine and lysine (23, 24), but is also critical for adverse pregnancy outcomes in a mouse model of preterm birth (24).

Recently, a genome-wide Tn5 transposon mutagenesis screen for identifying additional coaggregation factors revealed that the genetic disruption of a lysine metabolic pathway (LMP), e.g., deletion of *kamA* and *kamD* genes, blocks coaggregation through the excess accumulation of extracellular lysine, which binds and inhibits RadD (24). The subsequent discovery that the *kamA* deletion mutant is significantly attenuated in inducing preterm birth in mice (24) led to the realization that amino acid metabolism might play a key role in fusobacterial virulence. Consistent with this, targeted genetic analyses showed that a mutant, ∆*megL*, defective in cysteine/methionine metabolism leading to decreased hydrogen sulfide (H_2_S) production, is also attenuated in virulence in the mouse model of preterm birth (25). Yet another set of genes whose disruption by Tn5 insertional mutagenesis obliterated both coaggregation and biofilm development by fusobacteria encodes a putative respiratory enzyme commonly known as the Rnf (*Rhodobacter* nitrogen-fixation) complex (26).

Originally identified in *Rhodobacter capsulatus* (27), the Rnf complex is a highly conserved and evolutionarily ancient membrane-bound ferredoxin:NAD^+^ oxidoreductase that is thought to couple reversible electron transfer from reduced ferredoxin (fd_red_) to NAD^+^ with the establishment of an ion-motive force, enabling substrate import and/or ATP biosynthesis (27–29). Found in over 150 bacterial genomes and 2 archaeal genomes, with high occurrence in anaerobes (28), the Rnf complex has been demonstrated to be a multifunctional respiratory enzyme that acts as a versatile metabolic exchange center for nitrogen fixation (27), carbon dioxide fixation (30), metabolism of low-energy substrates, such as ethanol and lactate (31–33), gene regulation to some extent (34, 35), and gut colonization in mice (36). Importantly, whether the Rnf complex contributes to bacterial virulence remains to be explored. Here, we show that *F. nucleatum* encodes a functional *rnf* locus, *rnfC-rnfD-rnfG-rnfE-rnfA-rnfB*, and that genetic disruption of the Rnf complex, via deletion of *rnfC* or *rnfD*, causes severe defects in many virulence traits of *F. nucleatum*, including coaggregation, biofilm formation, H_2_S production, and ATP production, in addition to altering cell morphology and growth. Intriguingly, while the defect in coaggregation is mechanistically linked to a failure of lysine catabolism, leading to an increased level of extracellular lysine that inhibits RadD-mediated coaggregation, the defects in other traits are attributed to global reduction of amino acid metabolism as determined by targeted metabolomic analysis. Most significantly, the *rnfC* mutant is severely impaired in inducing preterm birth in mice. This study establishes that the Rnf complex is a central component in *F. nucleatum* metabolism that broadly impacts bacterial virulence.

## RESULTS

### Transposon mutagenesis reveals the involvement of the *F. nucleatum* Rnf complex in biofilm formation, coaggregation, and H_2_S production

**Fig 1:**
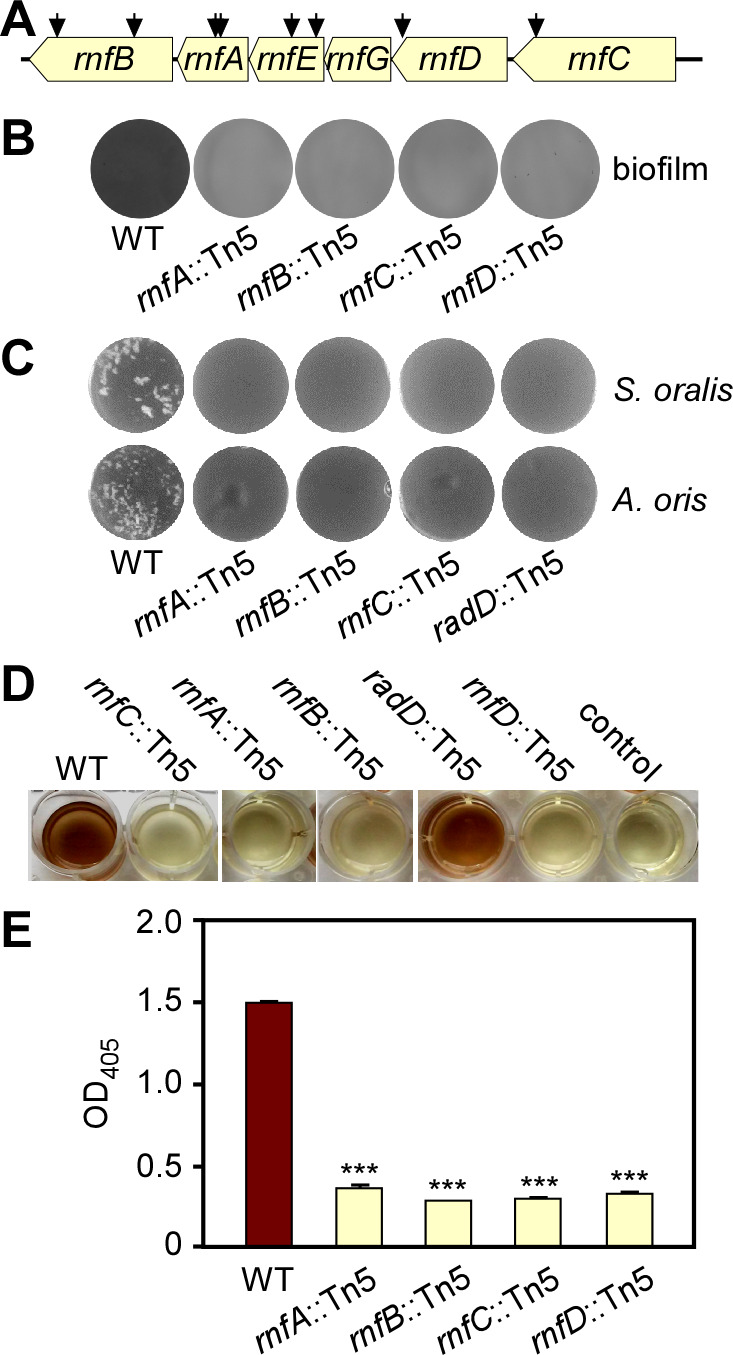
Transposon mutagenesis reveals the involvement of an Rnf complex in biofilm formation, coaggregation, and H_2_S production. (**A**) Shown is the *rnf* gene locus in *F. nucleatum*, with arrows indicating the Tn5 insertion sites of previously identified Tn5 mutants (24, 26). (**B**) Monospecies biofilms of the wild-type (WT) and Tn5 *rnf* mutant strains were cultivated in multi-well plates, stained with 1% crystal violet, and imaged. (**C**) Indicated fusobacterial strains were examined for their adherence to *S. oralis* and *A. oris* by a coaggregation assay. A *radD*::Tn5 mutant was used as a negative control. (**D–E**) Production of H_2_S from indicated strains was determined by the bismuth sulfide method (**D**) and quantified by absorbance measurement at 405 nm (**E**). Cell-free samples were used as a negative control. All results were obtained from three independent experiments performed in triplicate.

In our previous genome-wide screens that identified *F. nucleatum* mutants defective in biofilm formation and coaggregation, multiple subunit-encoding genes for the predicted Rnf complex were frequently targeted by Tn5 transposon insertion (24, 26) (Fig. 1A). To establish the potential multifunctional role of the Rnf complex, we further characterized these mutants in standard biofilm and coaggregation assays (24, 26). To cultivate monospecies biofilms, normalized fusobacterial cultures of different strains were seeded into sterile multi-well plates, anaerobically cultured at 37°C for 48 h, and the resultant biofilms were then stained with crystal violet. Compared to the wild-type strain (ATCC 23726), each of the four tested mutant strains were defective in biofilm formation (Fig. 1B). To examine whether these mutants are also deficient in coaggregation, normalized, unwashed fusobacterial cells were mixed and incubated with *Streptococcus oralis* or *Actinomyces oris* in a standard assay, followed by imaging (see Materials and Methods). The tested Tn5 mutants also failed to co-aggregate with these two oral co-colonizers at a level similar to that of a *radD* mutant used as a negative control (Fig. 1C).

Unexpectedly, we noticed that all of the *rnf* mutants lacked the rotten egg odor characteristically produced by wild-type fusobacteria, suggestive of severe deficiency in hydrogen sulfide production by these mutants. Indeed, in a bismuth sulfide assay, in which bismuth trichloride reacts with H_2_S to yield precipitation of brown bismuth sulfide, all *rnf*::Tn5 mutants showed a significantly reduced level of hydrogen sulfide relative to the wild-type strain (Fig. 1D and E). Overall, these results point to a multifaceted role of the Rnf complex in many cellular processes in *F. nucleatum*.

### Genetic deletion of *rnfC* disrupts RadD-mediated coaggregation via blockage of lysine catabolism

**Fig 2:**
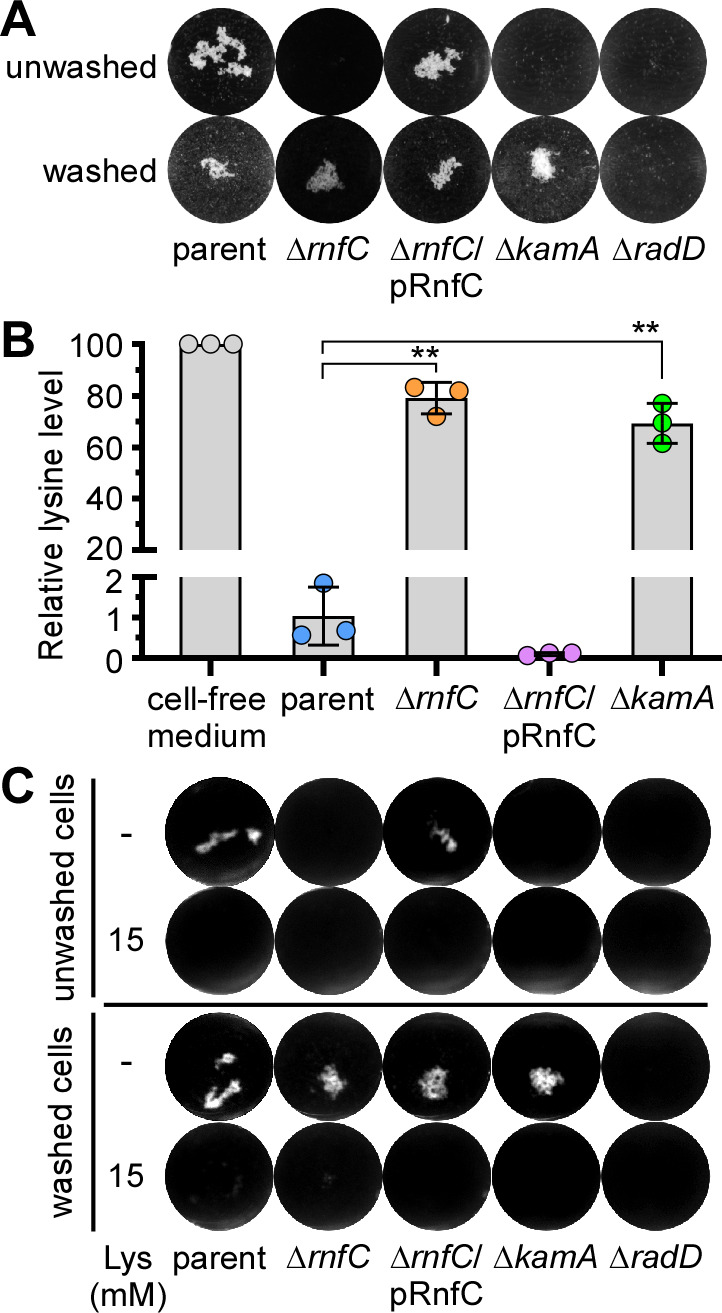
A non-polar, in-frame *rnfC* deletion mutant is defective in RadD-mediated coaggregation and lysine uptake. (**A**) The parent, *rnfC* deletion mutant, and *rnfC*-complemented strains were tested for their ability to co-aggregate with *S. gordonii*. Fusobacterial cells were washed or unwashed before being mixed in equal volumes with washed *S. gordonii* and imaged. Mutants devoid of *radD* or *kamA*, a lysine metabolic pathway gene (24), were used as references. (**B**) The relative level of lysine in the cell-free culture medium of indicated strains grown to stationary phase was determined by liquid chromatography-mass spectrometry, with the level of lysine in media without bacteria set to 100. (**C**) The same coaggregation experiment as (**A**) was performed with or without addition of 15 mM lysine. All results were obtained from three independent experiments performed in triplicate. Significance was calculated by a Student’s *t*-test; *P < 0.05; **P < 0.01.

A Tn5 insertion can potentially cause polar effects on transcription of downstream genes. Although this is unlikely the case here (Fig. S1A), we decided to generate non-polar, in-frame deletions of *rnf* genes utilizing our published protocols (24, 26, 37). Since all Tn5 mutants displayed similar phenotypes, we chose *rnfC* for further characterizations, as RnfC is predicted to be a peripheral membrane protein, enabling generation of polyclonal antibodies against a recombinant RnfC protein (Fig. S1B). To examine how the Rnf complex mediates polymicrobial interaction, this *rnfC* mutant (Δ*rnfC*) and other strains were subjected to the coaggregation assay, in which unwashed fusobacterial cells were mixed with *Streptococcus gordonii*, like the coaggregation assays done with *S. oralis* and *A. oris* as mentioned above (see Fig. 1C). As expected, deletion of *rnfC* abrogated fusobacterial coaggregation with *S. gordonii*, as compared to the parent strain (CW1), and this defect was rescued by ectopic expression of RnfC from a plasmid (Fig. 2A; the unwashed panel), supporting the polymicrobial nature of Rnf-associated coaggregation by *F. nucleatum* that is not limited to *S. oralis* and *A. oris*.

Because RadD is essential as the adhesin for fusobacterial coaggregation (23, 24), we examined if deletion of *rnfC* causes a defect in surface expression of RadD by immunofluorescence microscopy, wherein normalized fusobacterial cells of different strains were stained with antibodies against RadD (α-RadD), followed by counter-staining with Alexa 488-conjugated IgG. Microscopic examination presented in Fig. S2A revealed no significant defects of RadD-surface expression in the Δ*rnfC* mutant as compared to the parent strain. Consistent with this observation, western blotting of whole-cell lysates showed similar levels of RadD antigen production in all strains—parent, Δ*rnfC*, and its complement (Fig. S2B). Thus, the coaggregation defect of Δ*rnfC* is not due to reduced RadD expression.

Unexpectedly, when we utilized Δ*rnfC* cells free of the culture medium before mixing with *S. gordonii*, the mutant fusobacteria were able to adhere to oral streptococci at a level comparable to the parent strain (Fig. 2A; compare panels washed vs unwashed). This behavior of sensitivity to the culture medium mirrored that of a deletion mutant of *kamA*, which is defective in lysine catabolism and hence accumulates excess lysine in the culture medium (24). Given that lysine inhibits RadD-dependent coaggregation (23, 24), the results prompted us to examine the relative extracellular levels of lysine in these strains by using liquid chromatography-mass spectrometry as previously reported (24). Strikingly, compared to the parent strain, the extracellular level of lysine for the Δ*rnfC* mutant remained high and was quite comparable to that of the Δ*kamA* mutant (Fig. 2B). Thus, this accumulation of extracellular lysine is likely the inhibitor that blocks coaggregation by Δ*rnfC* mutant bacteria. To obtain support for this hypothesis, we re-performed the coaggregation assay with the same set of strains in the absence or presence of 15 mM lysine added in the coaggregation mix. Regardless of whether fusobacterial cells were washed or not, the addition of lysine blocked fusobacterial coaggregation with *S. gordonii* as expected (Fig. 2C).

Since deletion of *kamA* leads to the accumulation of extracellular lysine (Fig. 2B) (24), we examined the transcript level of *kamA* in the Δ*rnfC* mutant by quantitative reverse transcription polymerase chain reaction (qRT-PCR). Remarkably, expression of *kamA* was significantly reduced in the absence of *rnfC*, as compared to the parent strain, and this defect was rescued by ectopic expression of *rnfC* from a plasmid (Fig. S3). In addition, expression of *kamD*, another lysine metabolic gene in the *kamA* locus (24), mirrored that of *kamA* in these strains (Fig. S3). Altogether, the results indicate that the defect of RadD-mediated coaggregation by genetic disruption of *rnfC* is due to increased extracellular levels of lysine, likely due to the reduced expression of lysine metabolic genes by an as yet unexplored mechanism.

### Genetic deletion of *rnf*C disrupts biofilm formation, growth, cell morphology, and hydrogen sulfide production

**Fig 3:**
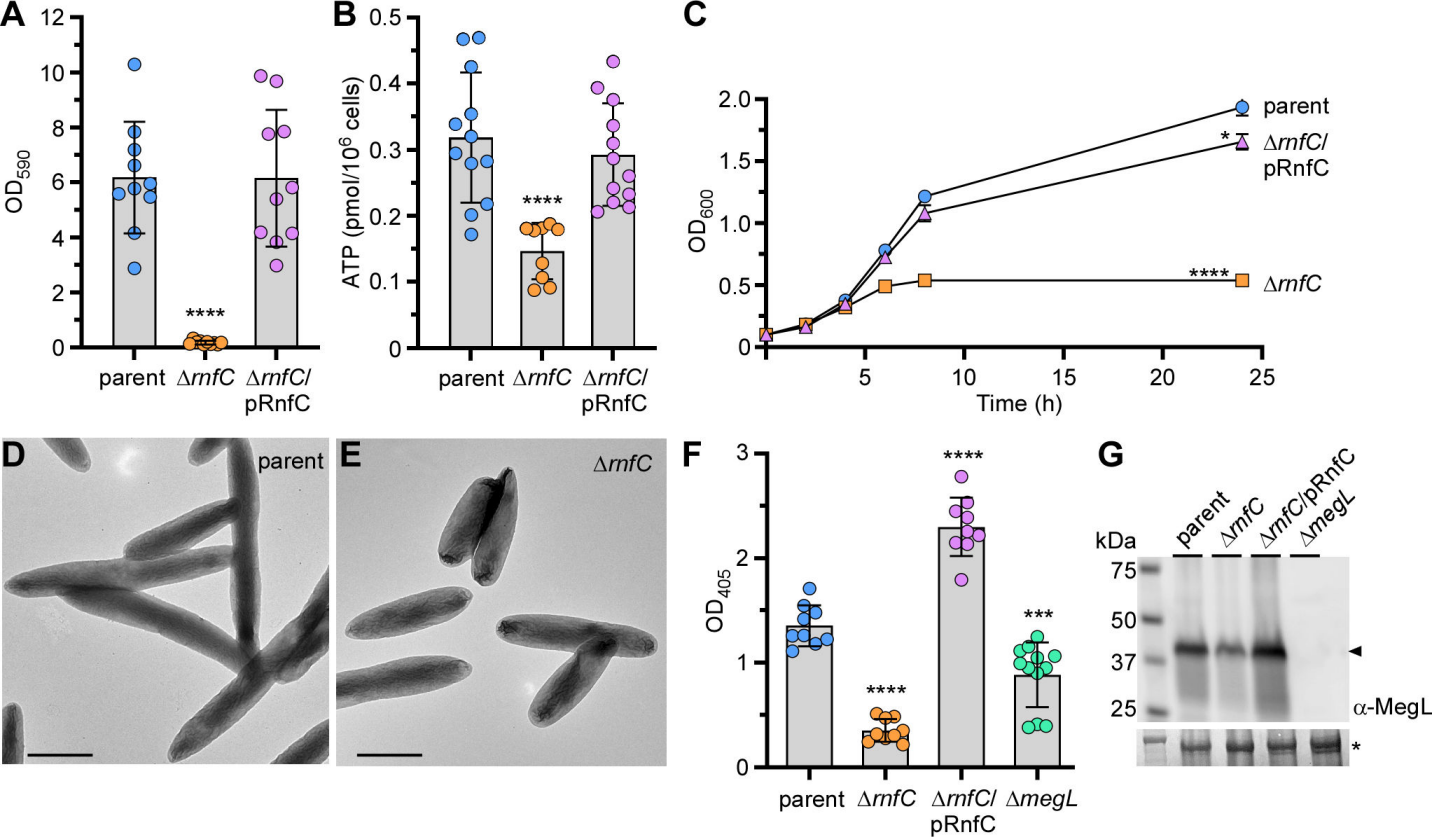
Deletion of *rnfC* causes pleiotropic defects. (**A**) 48-h grown biofilms of indicated strains were stained with 1% crystal violet and quantified by optical density measurement at 590 nm (OD_590_). (**B**) ATP production of indicated strains was assessed by a luciferase assay using a BacTiter-Glo kit (Promega). Mid-log phase cells were mixed with equal volumes of the BacTiter-Glo reagent and incubated at room temperature for 2 min before measuring luminescence on a plate reader. Normalized cell suspensions were serially diluted and plated for bacterial enumeration and subsequent normalization of relative luminescent signal. (**C**) Bacterial growth of indicated strains was determined by optical density at 600 nm at timed intervals. (**D–E**) Mid-log phase cells of parent and ∆*rnfC* mutant strains were immobilized on carbon-coated nickel grids and stained with 1% uranyl acetate prior to imaging by electron microscopy; scale bars: 1 µm. (**F**) H_2_S production of indicated strains was determined by the bismuth sulfide assay. (**G**) Protein samples obtained from the whole-cell lysates of normalized cultures of indicated strains were subjected to immunoblotting with antibodies against MegL (α-MegL). A Coomassie Blue stained band (*) was used as a loading control. All results were obtained from three independent experiments performed in triplicate. Significance was calculated by a Student’s *t*-test; *P < 0.05; ***P < 0.001; ****P < 0.0001.

Next, to determine how biofilm formation is dependent on the Rnf complex, we first analyzed the non-polar, in-frame *rnfC* deletion mutant in the aforementioned biofilm assay using crystal violet staining (as described in Fig. 1B above). As expected, this mutant was also unable to form monospecies biofilms compared to the parent strain, and ectopic expression of RnfC on a plasmid was sufficient to restore biofilm formation (Fig. 3A). Since the Rnf complex plays a key role in energy conservation through several bacterial metabolic pathways (27, 38, 39), we reasoned that the defects in biofilm formation by *rnf* mutants could be due to alterations with respect to fusobacterial physiology. To investigate this further, we tested whether the Rnf complex is important in ATP biosynthesis by subjecting normalized fusobacterial cells to a microbial cell viability assay (BacTiter-Glo), which relies on the mono-oxygenation of luciferin catalyzed by luciferase in the presence of ATP. Indeed, compared to the parent strain, the Δ*rnfC* mutant drastically reduced ATP production, and complementation with an RnfC-expressing plasmid rescued the defect (Fig. 3B). The Δ*rnfC* mutant was also severely defective in growth displaying premature growth cessation after the culture reached a suboptimal density (Fig. 3C). Intriguingly, the colony forming units (CFUs) of Δ*rnfC* cells grown to log phase and harvested for the ATP production assay or grown over the course of 24 h were comparable to that of the parent strain (Fig. S3). This similarity in the CFUs of both parent and Δ*rnfC* cells grown over time, although the latter shows reduced optical density, might be due to the morphological abnormality of the Δ*rnfC* mutant with short/stubby cells as examined by electron microscopy (Fig. 3D and E).

Potentially, the physiological deficits reported above might be due to reduced metabolism of amino acids, especially cysteine as it is one of eight important amino acids for fusobacteria (40). To test this hypothesis, we employed the aforementioned bismuth sulfide assay to measure hydrogen sulfide since it is a product of cysteine metabolism in *F. nucleatum* (25, 41–44). Consistent with the results of our Tn5 mutants, the Δ*rnfC* mutant showed a significant defect in H_2_S production, and overexpression of RnfC from a plasmid substantially enhanced this process (Fig. 3F). Because the methionine γ-lyase MegL was previously shown to be responsible for the bulk of H_2_S production from cysteine metabolism in *F. nucleatum* (25), we went on to determine the overall expression level of MegL in cells by immunoblotting of whole-cell lysates with antibodies against MegL (α-MegL). Remarkably, compared with the parent and rescued strains, the Δ*rnfC* mutant expressed a significantly reduced level of MegL (Fig. 3G). This reduction of MegL enzyme corresponded to a reduced level of *megL* mRNA as determined by qRT-PCR (Fig. S3). Notably, the expression of both *cysK1* and *cysK2*, which encode two additional H_2_S-producing enzymes (24), was also reduced in the absence of *rnfC* (Fig. S4).

To ascertain that the physiological defects reported above are a reflection of the loss of Rnf function, we repeated these experiments with a non-polar, in-frame *rnfD* deletion mutant (Δ*rnfD*). Like the Δ*rnfC* mutant, the Δ*rnfD* mutant showed significant defects in coaggregation, biofilm formation, cell growth, and H_2_S production, and these defects were rescued by ectopic expression of *rnfD* (Fig. S5). Altogether, these findings support our hypothesis that the biofilm formation defect caused by genetic disruption of the Rnf complex is not due to defects in cell viability or reduced cell numbers, but rather gross deficiencies in ATP biosynthesis and amino acid metabolism.

### Loss of RnfC complex disrupt global *F. nucleatum* metabolism

**Fig 4:**
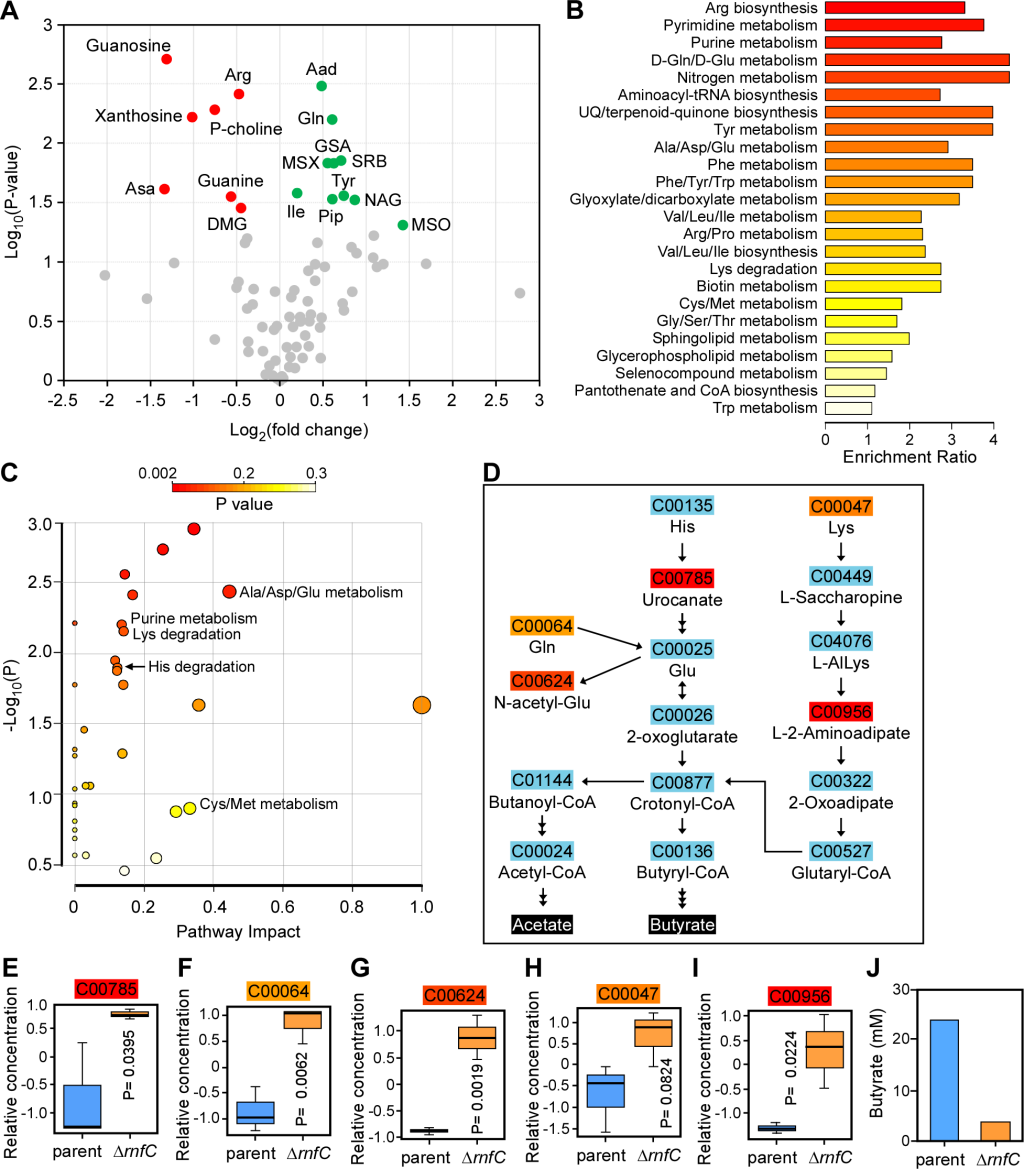
Deletion of *rnfC* disrupts amino acid metabolism. (**A**) The parent and Δ*rnfC* cells grown to mid-log phase were subjected to metabolomics analysis using liquid chromatography-mass spectrometry (LC-MS). Shown is a volcano plot of 81 differentially expressed metabolites (DEMs) between the parent and Δ*rnfC* mutant strains. Metabolites significantly depleted or enriched in the Δ*rnfC* strain, relative to the parent, are marked in red or green, respectively. (**B**) Shown is a graphical overview of quantitative enrichment analysis of DEMs between the parent and ∆*rnfC* strains generated using MetaboAnalyst 5.0. The top 24 pathway-associated metabolic sets in ∆*rnfC* compared to the parent strain were sorted based on their fold enrichment and *P*-values. (**C**) Pathway analysis was performed using MetaboAnalyst 5.0, which combines pathway enrichment and topology analysis. A range of *P*-values is shown from red to yellow. (**D**) Shown are amino acid metabolic nodes in *F. nucleatum*, based on the significance level in (**C**), generating acetate and butyrate. (**E–I**) The relative concentrations of DEMs in the amino acid nodes shown in (D) between the parent and Δ*rnfC* mutant strains are presented. *P*-values were calculated using the global test. (**J**) The relative level of butyrate (mM) in the overnight cultures of the parent and Δ*rnfC* strains was determined by LC-MS.

To gain further insight into the function of the *F. nucleatum* Rnf complex in relation to the aforementioned phenotypes associated with genetic disruption of the Rnf complex, we performed metabolomic analysis of parent and ∆*rnfC* cells using ultra-performance liquid chromatography coupled to mass spectrometry. Among over 80 metabolites detected in these samples, 10 metabolites (Gln, Tyr, Ile, etc.) were elevated, whereas 7 metabolites, including purine ribonucleoside precursors, were significantly depleted in the ∆*rnfC* mutant, as compared to the parent strain (Fig. 4A).

To obtain a better view of the specific metabolic pathways altered upon loss of *rnfC*, we performed a quantitative pathway enrichment and pathway topology analysis of the parent and ∆*rnfC* cell using the free, web-based software, MetaboAnalyst 5.0. In keeping with our initial findings (Fig. 4A), several pathways involved in amino acid and purine metabolism were significantly enriched in the ∆*rnfC* mutant compared to the parent strain (Fig. 4B and C). Pathway analysis of the “Lys degradation,” “His degradation,” and “Ala/Asp/Gln metabolism” nodes in the topology analysis revealed that many amino acids are directly degraded into glutamate, which is subsequently fermented into the short-chain fatty acids, butyrate, and acetate (Fig. 4D). Significantly, the ∆*rnfC* mutant had elevated levels of urocanate, glutamine, N-acetyl-L-glutamate, lysine, and the lysine-degradation pathway intermediate, L-2-aminoadipate, relative to the parent strain (Fig. 4E through I), indicating possible disruptions in glutamate fermentation. Consistent with this result, we observed a reduced level of butyrate in this mutant as determined by high-performance liquid chromatography coupled to mass spectrometry (Fig. 4J). Additionally, as demonstrated by pathway analysis of the “Cys/Met metabolism” node from the topology analysis, hydrogen sulfide production is derived primarily from methionine degradation into L-homocysteine and subsequent L-cysteine degradation into pyruvate (Fig. S6A), both catalyzed by MegL (25). Correspondingly, elevated levels of methionine and slightly lower levels of the pathway intermediate, S-adenosyl-L-homocysteine, were observed in the ∆*rnfC* mutant, relative to the parent strain (Fig. S6B and C), suggesting the Rnf complex may also be important for methionine/cysteine metabolism. In sum, the results support a broad role of the Rnf complex in *F. nucleatum* metabolism.

### The Rnf complex is required for *Fusobacterium nucleatum* virulence

**Fig 5:**
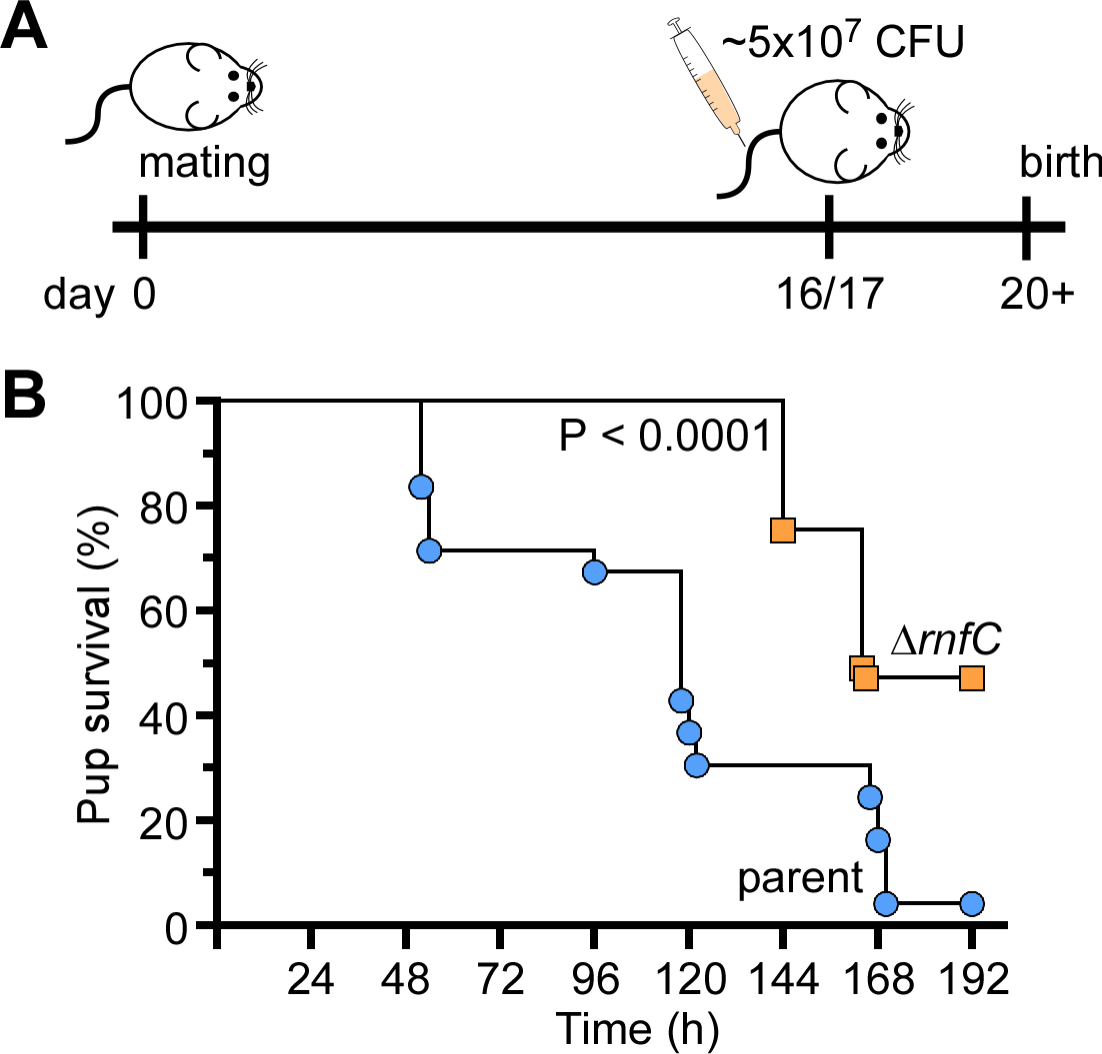
The *rnfC* mutant is attenuated in virulence. (**A–B**) Groups of pregnant CF-1 mice were infected with ~5.0 × 10^7^ CFU/mL of the parent or ∆*rnfC* strain via tail vein injection at day 16 or 17 of gestation. Pup survival was recorded over time. Statistical analysis was determined by Mantel-Cox testing.

Because genetic disruption of the Rnf complex causes significant defects in many virulence traits of *F. nucleatum* (Fig. 1 to 3), we sought to determine whether this complex contributes to *F. nucleatum* pathogenesis. Utilizing a mouse model of preterm birth as previously reported (24, 37), we infected via tail vein injection groups of five CF-1 pregnant mice at day 16/17 of gestation with roughly 5.0 × 10^7^ CFU of either the parent or ∆*rnfC* strain, and pup delivery was monitored (Fig. 5A). Strikingly, the ∆*rnfC* mutant was severely attenuated in virulence, with nearly 50% of pups born alive by the end-point, while almost no pups survived infection by the parent strain within the same timeframe (Fig. 5B). Clearly, the Rnf complex plays a significant role in metabolism that is central to *F. nucleatum* pathogenesis.

## DISCUSSION

The dual life of *F. nucleatum* as a commensal in the human oral cavity and an opportunistic pathogen in extra-oral sites raises an intriguing question as to how *F. nucleatum* is able to maintain its plasticity in metabolically changing environments. Here, we have demonstrated that the highly conserved multicomponent Rnf complex plays a central role in mediating metabolism of various amino acids in *F. nucleatum*, which impacts many physiological traits critical for fusobacterial virulence. Specifically, various *rnf* mutants emerged in unbiased forward genetic screens in which we sought to uncover genetic factors that govern two different aspects of *F. nucleatum* pathobiology—its ability to form biofilms and to co-aggregate with certain partner co-colonizers of the oral cavity. We then showed that that targeted disruption of the Rnf complex, via deletion of *rnfC* or *rnfD*, not only causes severe defects in coaggregation and biofilm formation but also hydrogen sulfide production, cell morphology and growth, ATP production, and induction of preterm birth (Fig. 1 to 5; Fig. S4).

The ability of fusobacteria to co-aggregate with streptococci and other partners such as *A. oris* depends on a well-characterized adhesin RadD present on the fusobacterial cell surface. We have shown that the loss of Rnf complex function does not alter RadD expression or its display at the bacterial surface (Fig. S2). Rather, the defect in RadD-mediated interbacterial coaggregation appears to be a consequence of a defect in lysine metabolism mediated by the LMP, which in turn raises the level of extracellular lysine that can bind and inhibit RadD to block coaggregation with oral streptococci and *A. oris* (Fig. 2). Intriguingly, our results revealed that this reduced lysine metabolism parallels a decreased expression of the LMP genes, i.e., *kamA* and *kamD* (Fig. S3). As KamA is predicted to catalyze the conversion of incoming lysine to β-lysine, which is then converted to 3,5-diaminohyxanoate by KamD (24), the reduced level of *kamA* and *kamD* caused by *rnfC* deletion would certainly contribute to the accumulation of extracellular lysine that inhibits coaggregation by binding to and inactivating the adhesin RadD (24). Not only is the Δ*rnfC* mutant unable to aggregate with oral bacteria, but it is also defective in biofilm formation. Because the viability of Δ*rnfC* cells remains comparable to the parent strain throughout growth phases (Fig. S3), although the optical density of the former is less than the parent during stationary phase growth (Fig. 3C) likely due to the reduced cell size (Fig. 3D and E), we think that the defect in biofilm formation is a result of defects in metabolism leading to reduced ATP production. It is noteworthy that the growth rate of *rnf* mutants is similar to that of the parent and complement strains during the early exponential growth before plateauing at the midpoint for the remainder of the growth period (Fig. 3C). It is likely that these mutants are able to utilize available sugars until their exhaustion but unable to metabolize various amino acids.

Consistently, our metabolomic profiling further revealed that the defect in amino acid metabolism is not limited to lysine alone as aforementioned but, rather, the metabolism of many additional amino acids (viz. cysteine, histidine, and glutamate) is also affected (Fig. 4; Fig. S6). Our targeted metabolomic analysis also revealed defects in purine metabolism, which is supported by significant reduction in guanine, adenine, and their respective nucleoside precursors in the absence of *rnfC* (Fig. 4A through C). Given that *F. nucleatum* harbors several purine nucleotide biosynthetic pathways originating from many amino acids such as glutamine, glutamate, and/or aspartate, it is likely that the defects in purine metabolism in the ∆*rnfC* mutant also result from aberrant amino acid utilization. Critically, cysteine metabolism that generates hydrogen sulfide in *F. nucleatum* is mainly catalyzed by MegL, a conserved L-methionine γ-lyase (25). Logically, we expected that the defective cysteine metabolism in the ∆*rnfC* mutant would lead to reduced hydrogen sulfide production by the mutant. What we did not anticipate, however, is that the level of hydrogen sulfide production in the ∆*rnfC* mutant is significantly lower than that of the *megL* mutant (Fig. 3F), indicating the Rnf complex plays a broader role in amino acid metabolism.

Indeed, pathway and gene expression analyses demonstrated that multiple pathways leading to hydrogen sulfide production are significantly affected in the absence of *rnfC* (Fig. S6) and that expression of other cysteine metabolic genes, e.g., *cysK1* and *cysK2*, is markedly reduced (Fig. S4). Intriguingly, given CysK1 catalyzes the conversion of cysteine to hydrogen sulfide and L-lanthionine, an essential amino acid required for formation of fusobacterial peptidoglycan (45, 46), the reduced expression of *cysK1* by deletion of *rnfC* can be attributed to aberrant cell growth, short and stumpy cell size, coupled with reduced ATP production (Fig. 3). How the absence of *rnfC* reduces expression of LMP genes, *megL*, and *cysK1*/*cysK2* genes remains a significant puzzle. It is noteworthy that the expression of LMP genes and *megL* is controlled by the two-component transduction systems (TCSs), CarRS and ModRS, respectively (24, 25). One intriguing possibility, which remains to be tested in future studies, is that metabolic blockage by genetic disruption of the Rnf complex causes the accumulation of certain metabolites and intermediates that may trigger gene expression responses from these TCSs and perhaps other transcriptional regulators.

**Fig 6:**
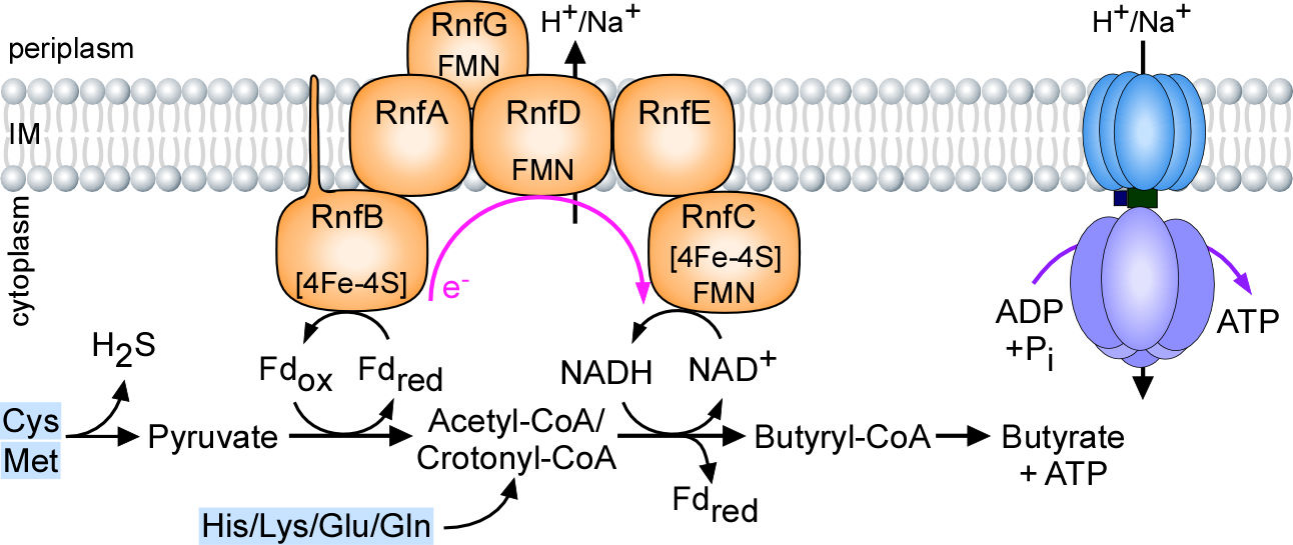
A working model of Rnf-mediated energy conservation through amino acid metabolism in *F. nucleatum*. See text for details.

How is the Rnf complex central to *F. nucleatum* metabolism and virulence? We propose that the *F. nucleatum* Rnf complex is a versatile metabolic exchange center that promotes metabolism of many amino acids, via the oxidation of reduced ferredoxin to the reduction of NAD^+^ that generates an electrochemical ion gradient across the cytoplasmic membrane, leading to the formation of several key metabolites and ATP (Fig. 6). In particular, cysteine and methionine are metabolized into H_2_S and pyruvate. The latter is oxidized to acetyl-CoA, leading to formation of butyryl-CoA and later butyrate, by reduction of ferredoxin (Fd_ox_) and NAD. Electron shuffling from fd_red_ to NAD+ by the Rnf complex generates a sodium/proton gradient that powers the ATP synthase to generate ATP. In addition, the fermentative pathways of many amino acids (His/Lys/Glu/Gln) also lead to crotonyl-CoA that is coupled to reduction of ferrodoxin. As such, genetic disruption of the Rnf complex limits the amino acid fermentative capacity of *F. nucleatum* causing pleiotropic defects that hinder the virulence capabilities of this pathobiont as seen through defects in coaggregation, biofilm formation, cell morphology and growth, H_2_S production, ATP biosynthesis, and induction of preterm birth. Given the wide conservation of the Rnf complex in many pathogens and its absence in eukaryotes, this multi-subunit respiratory enzyme may serve as an important target for broad anti-infective therapeutic strategies.

## MATERIALS AND METHODS

### Bacterial strains, plasmids, and media

The bacterial strains and plasmids used in this study are listed in Table S1. *F. nucleatum* cells were grown in tryptic soy broth supplemented with 1% Bacto peptone and 0.25% fresh cysteine (TSPC) or on TSPC agar plates in an anaerobic chamber (10% CO_2_, 10% H_2_, and 80% N_2_). Heart infusion broth or heart infusion agar was used to culture *A. oris* and supplemented with 0.5% glucose to grow *S. oralis* and *S. gordonii. Escherichia coli* strains were grown in Luria broth. All bacterial strains, except *F. nucleatum*, were cultured in a 5% CO_2_ incubator. When required, chloramphenicol or thiamphenicol were added into the medium at a concentration of 15 µg/mL and 5 µg/mL, respectively. All reagents were purchased from Sigma-Aldrich unless noted otherwise.

### Plasmid construction

(i) To generate pRnfC, the primer set com-rnfC-F/R (Table S2) was used to amplify the *rnfC* coding sequence and its promoter region from chromosomal DNA of *F. nucleatum* ATCC 23726, while appending the restriction sites KpnI/NdeI to the amplicon (35 cycles at annealing temperature of 55°C). The PCR product was digested with KpnI and NdeI restriction enzymes and cloned into pCWU6 (*SI* Table S2) precut with the same enzymes. (ii) To generate pRnfD, a segment encompassing the *rnfC* promoter region and the *rnfD* coding region was PCR-amplified from chromosomal DNA of *F. nucleatum* ∆*rnfC* (35 cycles at 56°C) using the primer set com-rnfD-F/R (Table S2), while appending NdeI and XhoI restriction sites to the amplicon. The PCR product was digested with NdeI and XhoI restriction enzymes and cloned into pCWU6 precut with the same enzymes. (iii) To generate pMCSG7-RnfC, the primer set, LIC-RnfC-F/R, was used to PCR-amplify the *rnfC* coding sequence from chromosomal DNA of *F. nucleatum* ATCC 23726, while appending adapter sequences for subsequent ligation-independent cloning into pMCSG7 as previously reported (47). All generated vectors were subjected to DNA sequence to confirm cloned sequences.

### Gene deletion in *F. nucleatum*

Generation of non-polar, in-frame deletion mutants, Δ*rnfC* and Δ*rnfD*, was performed according to our published protocol (24, 26, 37), with primers for generation of deletion constructs listed in Table S2. All PCRs were performed with a 55°C annealing temperature for 35 cycles.

### qRT-PCR

Overnight-grown (~17 h) fusobacterial cultures were normalized to optical density measurement at 600 nm (OD_600_) of ~2.0, and cells were harvested by centrifugation for RNA extraction using RNeasy Mini Kits (Qiagen) according to the manufacturer’s instructions, as described previously (25). Approximately 1 µg of purified RNA, free from DNA by treatment with DNase I (Qiagen), was used for cDNA synthesis using iScript RT supermix (Bio-Rad) based on the manufacturer protocol. Real-time PCR reactions were prepared using the SYBR Green PCR Master Mix with the appropriate primers (Table S2), and analysis was performed using the CFX96 Real-Time System (Bio-Rad). The ∆∆*C*_*T*_ method was used to calculate changes in gene expression between samples. Briefly, ∆∆*C*_*T*_ = ∆*C*_*T*1_ − ∆*C*_*T*2_, where ∆*C*_*T*_ = *C*_*T*_ (target) − *C*_*T*_ (housekeeping gene). Fold changes were calculated as log_10_(2^∆∆Ct^). The *16S* rRNA gene was used as reference. Reactions without reverse transcriptase were used as control to assess genomic DNA contamination.

### Biofilm assay

Stationary-phase (~20 h) cells of fusobacterial strains normalized to an OD_600_ of ~0.6 in fresh TSPC were seeded into flat-bottom, multi-well plates (Greiner Bio-One) and grown anaerobically for 48 h at 37°C. Obtained biofilms were washed gently in phosphate-buffered saline (PBS) and dried before being stained with 0.2 mL of 1% crystal violet (wt/vol) solution for 10 min at room temperature with gentle shaking. Biofilms were gently washed with sterile water before being dried and imaged. For quantification, dried biofilms were de-stained with acetic acid (30% vol/vol) for 10 min at room temperature, followed by measurement at OD_590_. Results were obtained from at least three independent experiments performed in triplicate. Statistical analysis was performed by GraphPad Prism 9.0.

### Bacterial coaggregation assays

Fusobacterial interaction with bacterial partner strains was assessed using a previously published coaggregation assay (24), with some modifications. Briefly, overnight cultures (~20 h) of *F. nucleatum* and partner strains (*S. oralis*, *S. gordonii*, and *A. oris*) were harvested by centrifugation and washed twice in coaggregation buffer (0.02 M tris-buffered saline, 150 mM NaCl, 0.1 mM CaCl_2_). Fusobacterial cells were normalized to an OD_600_ of ~0.4 in coaggregation buffer without or with 15 mM L-lysine, when indicated, whereas partner strains were normalized to an OD_600_ of 2.0 in coaggregation buffer. Also, 0.25 mL aliquots of normalized fusobacterial and partner strains were mixed in multi-well plates (GenClone), and coaggregation was imaged. For the experiments using unwashed fusobacterial cells, a similar procedure was used, except that fusobacterial cultures were directly used without washing. All coaggregation assays were performed in an aerated condition. Results were obtained from at least three independent experiments performed in triplicate.

### H_2_S detection

Detection of H_2_S was performed according to a published protocol with slight modification (25). Briefly, overnight cultures (~20 h) of fusobacterial cells were normalized to an OD_600_ of ~0.6 in TSPC, and 0.1 mL of normalized cell suspension was mixed with 0.1 mL of bismuth solution (0.4 M triethanolamine pH 8.0, 10 mM bismuth chloride, 20 mM pyridoxal 5-phosphate monohydride, 20 mM EDTA, 40 mM L-cysteine) and incubated at 37°C anaerobic for 1 h, at which point, images were taken and H_2_S was quantified by OD_405_. The results were presented as average of at least three independent experiments performed in triplicate.

### Measurement of lysine levels by liquid chromatography-mass spectrometry

The relative lysine levels from the culture supernatant of various fusobacterial strains grown to a stationary phase (~20 h) were determined at the Metabolomics Core at Baylor College of Medicine, according to our previous publication (24). Significance analysis was performed by GraphPad Prism.

### ATP quantification assay

Mid-log phase fusobacterial cells (OD_600_ of ~0.5) were harvested by centrifugation and combined in equal volumes (0.1 mL) with the BacTiter-Glo Reagent (Promega) in multi-well plates (Cellstar). The plates were incubated at room temperature in dark for ~2 min prior to measurement of relative luminescent unit using a plate reader (Tecan). ATP concentrations were determined from an ATP standard curve with ATP concentrations ranging from 1 nM to 1,000 nM. The results, presented as pmol/10^6^ CFU, were obtained from at least three independent experiments performed in triplicate.

### Quantification of CFU

For the ATP quantification assay, aliquots (0.1 mL) of fusobacterial cells harvested at mid-log phase and normalized to OD_600_ of 0.5 were serially diluted (10^0^–10^−7^) in a 96-well plate before spot plating 10 µL on TSPC agar plates supplemented with 5 µg/mL thiamphenicol when required. Plates were incubated in an anaerobic chamber at 37°C for 2 days before quantification. ATP levels of fusobacterial strains were normalized by their respective calculated CFU/mL. For CFU quantification over a 24-h growth period, stationary phase (~20 h) fusobacterial cultures were used to inoculate fresh cultures with starting OD_600_ of 0.1. Aliquots (0.1 mL) were taken at 8.5, 15, and 24 h of growth for spot plating and CFU/mL analysis. Results were obtained from three independent experiments performed in triplicate.

### Western blotting

Expression of fusobacterial proteins was analyzed by immunoblotting with antibodies against RadD (α-RadD; 1:5,000), MegL (α-MegL; 1:5,000), FtsX (α-FtsX; 1:2,000), and RnfC (α-RnfC; 1:3,000). Generation of the first three antibodies was described elsewhere (24–26). For α-RnfC, the procedure was followed according to a previously published protocol (26). Briefly, *E. coli* BL21 (DE3) harboring pMCSG7-RnfC was used to purify recombinant protein RnfC by affinity chromatography. The purified protein was used for antibody production (Cocalico Biologicals, Inc.). For immunoblotting, overnight fusobacterial cultures were normalized to an OD_600_ of 1.0, and 1 mL aliquots of bacterial cultures were taken for trichloroacetic acid precipitation and acetone wash, as previously reported (24). Protein samples obtained were suspended in SDS-containing sample buffer, separated by SDS-PAGE using 4%–15% Tris-Glycine gradient gels (RadD and MegL) or a 12% Tris-Glycine gel (RnfC), and immunoblotted by specific antibodies.

### Electron microscopy

Electron microscopy was performed according to previously published protocols (24, 26). Briefly, overnight cultures of fusobacteria were harvested by centrifugation and re-suspended in PBS supplemented with 0.1 M NaCl. A drop of bacterial suspension was added to carbon-coated grids, stained with 1% uranyl acetate, and washed prior to imaging with an electron microscope (JEOL 1200).

### Immunofluorescence microscopy

Overnight-grown (~20 h) fusobacterial cells were harvested by centrifugation and washed twice in PBS before normalizing to an OD_600_ of 0.5 in PBS. Also, 0.2 mL aliquots of cell suspension were used to coat circular glass coverslips placed in a 24-well plate for 20 min at room temperature. Cells were fixed with 2.5% formaldehyde (in PBS) for 20 min, washed with PBS, and blocked with 3% wt/vol bovine serum albumin for 1 h. Cells were incubated with α-RadD (1:200) for 1 h and then AlexaFluor 488 goat anti-rabbit IgG for another hour, followed by washing in PBS three times in the dark. Coverslips were mounted on glass slides with VECTASHIELD anti-fade mounting medium with DAPI (Vector Laboratories, Inc.). Cells were analyzed by a fluorescence microscope (Keyence BZ-X800).

### Targeted metabolic analysis

Parent and Δ*rnfC* mutant cells were harvested by centrifugation from mid-log phase cultures in triplicate and normalized to an OD_600_ of 0.5 before drying in a speed vacuum concentrator (Thermo Scientific). Dried pellet samples were subjected to metabolomics analysis at the UC Riverside Metabolomics Core Facility as previously described with some modifications (48). In brief, 10 mg of dry pellet was extracted with 100 µL/mg of monophasic extraction solvent (30:30:20:20/acetonitrile, methanol, isopropanol, and water) by sonication on ice for 5 min, followed by 90-min vortexing at 4°C. Cell-free supernatants were collected by centrifugation (3,000 *g*) at 4°C for 30 min, and 1 mL aliquots were analyzed by a TQ-XS triple quadrupole mass spectrometer (Waters) coupled to an I-class UPLC system (Waters), with separations performed using a ZIC-pHILIC column (2.1 × 150 mm, 5 µM) (EMD Millipore). The mobile phases were (A) water supplemented with 15 mM ammonium bicarbonate titrated to a pH of 9.6 with ammonium hydroxide and (B) acetonitrile. The flow rate was 200 µL/min, and the columns were held at 50°C. The injection volume was 2 µL. The gradient was as follows: 0 min, 90% B; 1.5 min, 90% B; 16 min, 20% B; 18 min, 20% B; 20 min, 90% B; 28 min, 90% B. The MS was operated in multiple-reaction monitoring mode. Source and desolvation temperatures were 150°C and 600°C, respectively. Desolvation gas was set to 1,100 L/h and cone gas to 150 L/h. Collision gas was set to 0.15 mL/min. All gases were nitrogen except the collision gas of argon. Capillary voltage was 1 kV in positive ion mode and 2 kV in negative ion mode. Four quality control (QC) samples, generated by pooling equal aliquots of each sample, were analyzed periodically to monitor system stability and performance. Samples were analyzed in random order. Statistical analysis was performed relative to the parent strain and determined by a Student’s *t*-test, using GraphPad Prism 9.0.

Raw mass spectral data for all 81 identified metabolites with corresponding Kyoto Encyclopedia of Genes and Genomes (KEGG) identifiers above for *F. nucleatum* ATCC 23726 were used to perform quantitative enrichment and pathway analysis using MetaboAnalyst 5.0 (49, 50). Statistical analysis was performed relative to the parent strain and determined by the global test of MetaboAnalyst 5.0. The raw metabolomic data can be accessed from Github with the following link: https://github.com/Timbritton7/RawMetabolomicAnalysis_Britton.git.

### Mouse model of preterm birth

The virulence potential of Δ*rnfC* was evaluated using a published mouse model of preterm birth (25, 37). Briefly, groups of five CF-1 (Charles River Laboratories) pregnant mice were infected with ~5 × 10^7^ CFU of the parent or ∆*rnfC* strain at day 16 or 17 of gestation via tail vein injection. Pup survival was recorded for the next 7 days. Statistical analysis was determined via the Mantel-Cox text, using GraphPad Prism 9.0, and specified in corresponding figure legends.

## SUPPLEMENTAL MATERIAL

Supplemental material mbio.01751-23-s0001.pdf Supplemental figures and tables.

ASM does not own the copyrights to Supplemental Material that may be linked to, or accessed through, an article. The authors have granted ASM a non-exclusive, world-wide license to publish the Supplemental Material files. Please contact the corresponding author directly for reuse.

## References

[1] Dewhirst FE, Chen T, Izard J, Paster BJ, Tanner ACR, Yu W-H, Lakshmanan A, Wade WG. 2010. The human oral microbiome. J Bacteriol 192:5002–5017. doi:10.1128/JB.00542-10 20656903 PMC2944498

[2] Caselli E, Fabbri C, D’Accolti M, Soffritti I, Bassi C, Mazzacane S, Franchi M. 2020. Defining the oral microbiome by whole-genome sequencing and resistome analysis: the complexity of the healthy picture. BMC Microbiol 20:120. doi:10.1186/s12866-020-01801-y 32423437 PMC7236360

[3] Kolenbrander PE, Andersen RN, Moore LV. 1989. Coaggregation of Fusobacterium nucleatum, Selenomonas flueggei, Selenomonas infelix, Selenomonas noxia, and Selenomonas sputigena with strains from 11 genera of oral bacteria. Infect Immun 57:3194–3203. doi:10.1128/iai.57.10.3194-3203.1989 2777378 PMC260789

[4] Lancy P, Dirienzo JM, Appelbaum B, Rosan B, Holt SC. 1983. Corncob formation between Fusobacterium nucleatum and Streptococcus sanguis. Infect Immun 40:303–309. doi:10.1128/iai.40.1.303-309.1983 6131871 PMC264849

[5] Coppenhagen-Glazer S, Sol A, Abed J, Naor R, Zhang X, Han YW, Bachrach G. 2015. Fap2 of Fusobacterium nucleatum is a galactose-inhibitable adhesin involved in coaggregation, cell adhesion, and preterm birth. Infect Immun 83:1104–1113. doi:10.1128/IAI.02838-14 25561710 PMC4333458

[6] Mutha NVR, Mohammed WK, Krasnogor N, Tan GYA, Choo SW, Jakubovics NS. 2018. Transcriptional responses of Streptococcus gordonii and Fusobacterium nucleatum to coaggregation. Mol Oral Microbiol 33:450–464. doi:10.1111/omi.12248 30329223

[7] Wu T, Cen L, Kaplan C, Zhou X, Lux R, Shi W, He X. 2015. Cellular components mediating coadherence of Candida albicans and Fusobacterium nucleatum. J Dent Res 94:1432–1438. doi:10.1177/0022034515593706 26152186 PMC4577983

[8] Rickard AH, Gilbert P, High NJ, Kolenbrander PE, Handley PS. 2003. Bacterial coaggregation: an integral process in the development of multi-species biofilms. Trends Microbiol 11:94–100. doi:10.1016/s0966-842x(02)00034-3 12598132

[9] Kolenbrander PE, Andersen RN. 1989. Inhibition of coaggregation between Fusobacterium nucleatum and Porphyromonas (Bacteroides) gingivalis by lactose and related sugars. Infect Immun 57:3204–3209. doi:10.1128/iai.57.10.3204-3209.1989 2777379 PMC260790

[10] Karched M, Bhardwaj RG, Asikainen SE. 2015. Coaggregation and biofilm growth of granulicatella spp. with Fusobacterium nucleatum and Aggregatibacter actinomycetemcomitans. BMC Microbiol 15:114. doi:10.1186/s12866-015-0439-z 26025449 PMC4448563

[11] Polak D, Shapira L, Weiss EI, Houri-Haddad Y. 2012. The role of coaggregation between Porphyromonas gingivalis and Fusobacterium nucleatum on the host response to mixed infection. J Clin Periodontol 39:617–625. doi:10.1111/j.1600-051X.2012.01889.x 22607053

[12] Yamaguchi-Kuroda Y, Kikuchi Y, Kokubu E, Ishihara K. 2023. Porphyromonas gingivalis diffusible signaling molecules enhance Fusobacterium nucleatum biofilm formation via gene expression modulation. J Oral Microbiol 15:2165001. 36687169 10.1080/20002297.2023.2165001 PMC9848294

[13] Han YW, Shen T, Chung P, Buhimschi IA, Buhimschi CS. 2009. Uncultivated bacteria as etiologic agents of intra-amniotic inflammation leading to preterm birth. J Clin Microbiol 47:38–47. doi:10.1128/JCM.01206-08 18971361 PMC2620857

[14] Payne MS, Bayatibojakhi S. 2014. Exploring preterm birth as a polymicrobial disease: an overview of the uterine microbiome. Front Immunol 5:595. doi:10.3389/fimmu.2014.00595 25505898 PMC4245917

[15] Parhi L, Alon-Maimon T, Sol A, Nejman D, Shhadeh A, Fainsod-Levi T, Yajuk O, Isaacson B, Abed J, Maalouf N, Nissan A, Sandbank J, Yehuda-Shnaidman E, Ponath F, Vogel J, Mandelboim O, Granot Z, Straussman R, Bachrach G. 2020. Breast cancer colonization by Fusobacterium nucleatum accelerates tumor growth and metastatic progression. Nat Commun 11:3259. doi:10.1038/s41467-020-16967-2 32591509 PMC7320135

[16] Udayasuryan B, Ahmad RN, Nguyen TTD, Umaña A, Monét Roberts L, Sobol P, Jones SD, Munson JM, Slade DJ, Verbridge SS. 2022. Fusobacterium nucleatum induces proliferation and migration in pancreatic cancer cells through host autocrine and paracrine signaling. Sci Signal 15:eabn4948. doi:10.1126/scisignal.abn4948 36256708 PMC9732933

[17] Rubinstein MR, Wang X, Liu W, Hao Y, Cai G, Han YW. 2013. Fusobacterium nucleatum promotes colorectal carcinogenesis by modulating E-cadherin/beta-catenin signaling via its FadA adhesin. Cell Host Microbe 14:195–206. doi:10.1016/j.chom.2013.07.012 23954158 PMC3770529

[18] Abed J, Emgård JEM, Zamir G, Faroja M, Almogy G, Grenov A, Sol A, Naor R, Pikarsky E, Atlan KA, Mellul A, Chaushu S, Manson AL, Earl AM, Ou N, Brennan CA, Garrett WS, Bachrach G. 2016. Fap2 mediates Fusobacterium nucleatum colorectal adenocarcinoma enrichment by binding to tumor-expressed gal-GaLNAc. Cell Host Microbe 20:215–225. doi:10.1016/j.chom.2016.07.006 27512904 PMC5465824

[19] Parhi L, Abed J, Shhadeh A, Alon-Maimon T, Udi S, Ben-Arye SL, Tam J, Parnas O, Padler-Karavani V, Goldman-Wohl D, Yagel S, Mandelboim O, Bachrach G. 2022. Placental colonization by Fusobacterium nucleatum is mediated by binding of the Fap2 lectin to placentally displayed Gal-GaLNAc. Cell Rep 38:110537. doi:10.1016/j.celrep.2022.110537 35320712

[20] Rubinstein MR, Baik JE, Lagana SM, Han RP, Raab WJ, Sahoo D, Dalerba P, Wang TC, Han YW. 2019. Fusobacterium nucleatum promotes colorectal cancer by inducing Wnt/beta-catenin modulator annexin A1. EMBO Rep 20:e47638. doi:10.15252/embr.201847638 30833345 PMC6446206

[21] Xu M, Yamada M, Li M, Liu H, Chen SG, Han YW. 2007. FadA from Fusobacterium nucleatum utilizes both secreted and nonsecreted forms for functional oligomerization for attachment and invasion of host cells. J Biol Chem 282:25000–25009. doi:10.1074/jbc.M611567200 17588948

[22] Ikegami A, Chung P, Han YW. 2009. Complementation of the fadA mutation in Fusobacterium nucleatum demonstrates that the surface-exposed Adhesin promotes cellular invasion and placental colonization. Infect Immun 77:3075–3079. doi:10.1128/IAI.00209-09 19398541 PMC2708594

[23] Kaplan CW, Lux R, Haake SK, Shi W. 2009. The Fusobacterium nucleatum outer membrane protein RadD is an arginine-inhibitable adhesin required for inter-species adherence and the structured architecture of multispecies biofilm. Mol Microbiol 71:35–47. doi:10.1111/j.1365-2958.2008.06503.x 19007407 PMC2741168

[24] Wu C, Chen Y-W, Scheible M, Chang C, Wittchen M, Lee JH, Luong TT, Tiner BL, Tauch A, Das A, Ton-That H. 2021. Genetic and molecular determinants of polymicrobial interactions in Fusobacterium nucleatum. Proc Natl Acad Sci U S A 118:e2006482118. doi:10.1073/pnas.2006482118 34074747 PMC8201914

[25] Chen Y-W, Camacho MI, Chen Y, Bhat AH, Chang C, Peluso EA, Wu C, Das A, Ton-That H. 2022. Genetic determinants of hydrogen sulfide biosynthesis in Fusobacterium Nucleatum are required for bacterial fitness, antibiotic sensitivity, and virulence. mBio 13:e0193622. doi:10.1128/mbio.01936-22 36073813 PMC9600241

[26] Wu C, Al Mamun AAM, Luong TT, Hu B, Gu J, Lee JH, D’Amore M, Das A, Ton-That H. 2018. Forward genetic dissection of biofilm development by Fusobacterium nucleatum: novel functions of cell division proteins FtsX and EnvC. mBio 9:e00360-18. doi:10.1128/mBio.00360-18 29691334 PMC5915739

[27] Schmehl M, Jahn A, Meyer zu Vilsendorf A, Hennecke S, Masepohl B, Schuppler M, Marxer M, Oelze J, Klipp W. 1993. Identification of a new class of nitrogen fixation genes in Rhodobacter capsulatus: a putative membrane complex involved in electron transport to nitrogenase. Mol Gen Genet 241:602–615. doi:10.1007/BF00279903 8264535

[28] Reyes-Prieto A, Barquera B, Juárez O. 2014. Origin and evolution of the sodium -pumping NADH: ubiquinone oxidoreductase. PLoS One 9:e96696. doi:10.1371/journal.pone.0096696 24809444 PMC4014512

[29] Biegel E, Müller V. 2010. Bacterial Na+-translocating ferredoxin:NAD+ oxidoreductase. Proc Natl Acad Sci U S A 107:18138–18142. doi:10.1073/pnas.1010318107 20921383 PMC2964206

[30] Biegel E, Schmidt S, Müller V. 2009. Genetic, immunological and biochemical evidence for a Rnf complex in the acetogen Acetobacterium woodii. Environ Microbiol 11:1438–1443. doi:10.1111/j.1462-2920.2009.01871.x 19222539

[31] Neumann-Schaal M, Jahn D, Schmidt-Hohagen K. 2019. Metabolism the difficile way: the key to the success of the pathogen Clostridioides difficile. Front Microbiol 10:219. doi:10.3389/fmicb.2019.00219 30828322 PMC6384274

[32] Weghoff MC, Bertsch J, Müller V. 2015. A novel mode of lactate metabolism in strictly anaerobic bacteria. Environ Microbiol 17:670–677. doi:10.1111/1462-2920.12493 24762045

[33] Seedorf H, Fricke WF, Veith B, Brüggemann H, Liesegang H, Strittmatter A, Miethke M, Buckel W, Hinderberger J, Li F, Hagemeier C, Thauer RK, Gottschalk G. 2008. The genome of Clostridium kluyveri, a strict anaerobe with unique metabolic features. Proc Natl Acad Sci U S A 105:2128–2133. doi:10.1073/pnas.0711093105 18218779 PMC2542871

[34] Hreha TN, Mezic KG, Herce HD, Duffy EB, Bourges A, Pryshchep S, Juarez O, Barquera B. 2015. Complete topology of the RNF complex from Vibrio cholerae. Biochemistry 54:2443–2455. doi:10.1021/acs.biochem.5b00020 25831459 PMC5019954

[35] Koo MS, Lee JH, Rah SY, Yeo WS, Lee JW, Lee KL, Koh YS, Kang SO, Roe JH. 2003. A reducing system of the superoxide sensor SoxR in Escherichia coli*.* EMBO J 22:2614–2622. doi:10.1093/emboj/cdg252 12773378 PMC156749

[36] Liu Y, Chen H, Van Treuren W, Hou B-H, Higginbottom SK, Dodd D. 2022. Clostridium sporogenes uses reductive stickland metabolism in the gut to generate ATP and produce circulating metabolites. Nat Microbiol 7:695–706. doi:10.1038/s41564-022-01109-9 35505245 PMC9089323

[37] Peluso EA, Scheible M, Ton-That H, Wu C. 2020. Genetic manipulation and virulence assessment of Fusobacterium nucleatum. Curr Protoc Microbiol 57:e104. doi:10.1002/cpmc.104 32539234 PMC7398570

[38] Biegel E, Schmidt S, González JM, Müller V. 2011. Biochemistry, evolution and physiological function of the Rnf complex, a novel ion-motive electron transport complex in prokaryotes. Cell Mol Life Sci 68:613–634. doi:10.1007/s00018-010-0555-8 21072677 PMC11115008

[39] Tremblay P-L, Zhang T, Dar SA, Leang C, Lovley DR, Newman DK. 2013. The Rnf complex of Clostridium ljungdahlii is a proton-translocating ferredoxin:NAD+ oxidoreductase essential for autotrophic growth. mBio 4:e00406-12. doi:10.1128/mBio.00406-12 PMC3531802 23269825

[40] Dzink JL, Socransky SS. 1990. Amino acid utilization by Fusobacterium nucleatum grown in a chemically defined medium. Oral Microbiol Immunol 5:172–174. doi:10.1111/j.1399-302x.1990.tb00418.x 2080074

[41] Fukamachi H, Nakano Y, Yoshimura M, Koga T. 2002. Cloning and characterization of the L-cysteine desulfhydrase gene of Fusobacterium nucleatum. FEMS Microbiol Lett 215:75–80. doi:10.1111/j.1574-6968.2002.tb11373.x 12393204

[42] Yoshida Y, Suwabe K, Nagano K, Kezuka Y, Kato H, Yoshimura F. 2011. Identification and enzymic analysis of a novel protein associated with production of hydrogen sulfide and L-serine from L-cysteine in Fusobacterium nucleatum subsp. nucleatum ATCC 25586. Microbiology (Reading) 157:2164–2171. doi:10.1099/mic.0.048934-0 21493682

[43] Yoshida Y, Ito S, Kamo M, Kezuka Y, Tamura H, Kunimatsu K, Kato H. 2010. Production of hydrogen sulfide by two enzymes associated with biosynthesis of homocysteine and lanthionine in Fusobacterium nucleatum subsp. Microbiology (Reading) 156:2260–2269. doi:10.1099/mic.0.039180-0 20413556

[44] Suwabe K, Yoshida Y, Nagano K, Yoshimura F. 2011. Identification of an L-methionine gamma-lyase involved in the production of hydrogen sulfide from L-cysteine in Fusobacterium nucleatum subsp. nucleatum ATCC 25586. Microbiology (Reading) 157:2992–3000. doi:10.1099/mic.0.051813-0 21798982

[45] Vasstrand EN, Hofstad T, Endresen C, Jensen HB. 1979. Demonstration of lanthionine as a natural constituent of the peptidoglycan of Fusobacterium nucleatum. Infect Immun 25:775–780. doi:10.1128/iai.25.3.775-780.1979 500186 PMC414514

[46] Fredriksen A, Vasstrand EN, Jensen HB. 1991. Peptidoglycan precursor from Fusobacterium nucleatum contains lanthionine. J Bacteriol 173:900–902. doi:10.1128/jb.173.2.900-902.1991 1987169 PMC207087

[47] Siegel SD, Amer BR, Wu C, Sawaya MR, Gosschalk JE, Clubb RT, Ton-That H. 2019. Structure and mechanism of LcpA, a phosphotransferase that mediates glycosylation of a gram-positive bacterial cell wall-anchored protein. mBio 10:e01580-18. doi:10.1128/mBio.01580-18 PMC6381275 30782654

[48] Vliet SMF, Dasgupta S, Sparks NRL, Kirkwood JS, Vollaro A, Hur M, Zur Nieden NI, Volz DC. 2019. Maternal-to-zygotic transition as a potential target for niclosamide during early embryogenesis. Toxicol Appl Pharmacol 380:114699. doi:10.1016/j.taap.2019.114699 31398420 PMC6717554

[49] Xia J, Psychogios N, Young N, Wishart DS. 2009. Metaboanalyst: a web server for metabolomic data analysis and interpretation. Nucleic Acids Res 37:W652–60. doi:10.1093/nar/gkp356 19429898 PMC2703878

[50] Pang Z, Zhou G, Ewald J, Chang L, Hacariz O, Basu N, Xia J. 2022. Using MetaboAnalyst 5.0 for LC-HRMS spectra processing, multi-omics integration and covariate adjustment of global metabolomics data. Nat Protoc 17:1735–1761. doi:10.1038/s41596-022-00710-w 35715522

