# Supplementary material for: The respiratory enzyme complex Rnf is vital for metabolic adaptation and virulence in Fusobacterium nucleatum
Source: mBio. 2023 Dec 7;15(1):e01751-23. doi: 10.1128/mbio.01751-23 (PMC10790702; doi:10.1128/mbio.01751-23)
Supplement: Supplemental material — Supplemental figures and tables. [file mbio.01751-23-s0001.pdf]

# The respiratory enzyme complex Rnf is vital for metabolic adaptation and virulence in *Fusobacterium nucleatum*

**Timmie A. Britton[a], Chenggang Wu[b], Yi-Wei Chen[c], Dana Franklin[a], Yimin Chen[c], Martha I. Camacho[c], Truc T. Luong[c], Asis Das[d], and Hung Ton-That[a,c,e,1]**

[a]*Molecular Biology Institute, University of California, Los Angeles, California, USA;* [b]*Department of Microbiology & Molecular Genetics, University of Texas McGovern Medical School, Houston, Texas, USA;* [c]*Division of Oral & Systemic Health Sciences,, School of Dentistry, University of California, Los Angeles, California, USA;* [d]*Department of Medicine, Neag Comprehensive Cancer Center, University of Connecticut Health Center, Farmington, CT, USA;* [e]*Department of Microbiology, Immunology & Molecular Genetics, University of California, Los Angeles, Los Angeles, CA, USA.*

**[1]**To whom correspondence may be addressed; email htonthat@dentistry.ucla.edu

Running Title: Rnf complex in bacterial virulence

Keywords: Rnf complex, metabolism, hydrogen sulfide, coaggregation, preterm birth, virulence

## Supporting Figures

**Figure S1: Expression analysis of RnfC by western blotting. (A)** Protein samples in the whole-cell lysates prepared from the parent and Tn5 mutant strains were subjected to SDS-PAGE and immunoblotted with antibodies against RnfC (α-RnfC). **(B)** Immunoblotting of whole-cell lysates of the parent, its isogenic *rnfC* deletion mutant Δ*rnfC*, and *rnfC*-complemented Δ*rnfC*/pRnfC strains was performed with α-RnfC. Blotting with antibody against FtsX (the membrane-bound cell-division protein) was used as the loading control. Results represent three independent experiments performed in triplicate.

**Figure S2: RnfC is dispensable for surface display of RadD. (A)** Overnight-grown cells of indicated fusobacterial strains were first stained with an antibody against RadD (α-RadD) and then Alexa Fluor 488-conjugated secondary antibody (green), as well as DAPI (blue). Surface display of RadD was analyzed by fluorescence microscopy. **(B)** Expression of RadD was analyzed by immunoblotting of protein samples in the whole-cell lysates obtained from the indicated strains. A Coomassie-stained band (*) was used as loading control. The results presented are representative of three independent experiments performed in triplicate.

**Figure S3: Colony forming units of the Δ*rnfC* mutant are comparable to the parent strain. (A)** Normalized overnight cultures of the parent, Δ*rnfC*, complementing strains were used to inoculate fresh cultures grown to mid-log phase ($OD_{600}$ of 0.5). Aliquots were taken for ATP quantification (see Fig. 3B) and bacterial numeration expressed as colony forming unit per ml (CFU/ml). **(B)** Normalized overnight cultures of indicated strains were used to inoculate fresh cultures that were grown for 24. Aliquots were taken at indicated times for bacterial numeration (CFU/ml). Results represent three independent experiments performed in triplicate. Significance was calculated by a student's t-test; *$P < 0.05$; **$P < 0.01$; ***$P < 0.001$.

**Figure S4: Deletion of *rnfC* significantly reduces expression of genes coding for enzymes involved in $H_2S$ production and lysine catabolism.** Normalized overnight cultures of the parent, in Δ*rnfC*, and Δ*rnfC*/pRnfC strains were used to isolate total RNA. The expression levels of *megL*, *cysK1*, *cysK2*, *kamA*, *kamD*, and *radD* in these strains were determined by qRT-PCR. Results were obtained from three independent experiments performed in triplicate. All qRT-PCR data were normalized to the transcript abundance of 16s rRNA for each sample. Significance was calculated by a student's t-test; ****, P < 0.0001.

**Figure S5: Deletion of *rnfD* causes pleiotropic defects. (A)** Interaction between *S. gordonii* DL1 and indicated fusobacterial strains was determined by a coaggregation assay, with fusobacterial cells washed or unwashed prior to mixing with *gordonii*. A *radD* mutant was used as a negative control. **(B)** Bacterial growth of indicated strains was monitored by optical density at 600 nm over 24 h. **(C)** Biofilms of indicated strains were cultivated for 48 h under anaerobic conditions. Quantification of biofilm production was determined by 1% crystal violet staining. **(D)** Normalized overnight cultures of indicated fusobacterial strains were used to determine hydrogen sulfide production by a bismuth assay. Results represent three independent experiments performed in triplicate, and significance calculated by a student's t-test; ****, P< 0.0001.

**Figure S6: Deletion of *rnfC* alters methionine and cysteine metabolism. (A)** Using the MetaboAnalyst 5.0 web-based software, pathway analysis was performed on differentially expressed metabolites between the parent and Δ*rnfC* strain (see Fig. 4C). Shown is the generated pathway from the "Cys/Met metabolism" node. The level of significance for a metabolite abundantly detected in these two strains is indicated by yellow to red (P value ranging from 0.3 to 0.002). KEGG identification numbers of metabolites are highlighted in light blue. **(B-C)** Shown are relative concentrations of methionine (B) and S-adenosyl-L-homocysteine (C); statistical significance was calculated using the Global test.

**Supporting Tables**

**Table S1: Bacterial strains and plasmids used in this study**

| Strains & Plasmids | Description | Reference |
|---|---|---|
| *Strain* | | |
| *F. nucleatum* ATCC 23726 | Type strain | (1) |
| *F. nucleatum* CW1 | Derivative of 23726; lacking *galK* (Δ*galK*) | (1) |
| *F. nucleatum radD*::Tn5 | Derivative of ATCC 23726 with Tn5 insertion into *radD* at position 5615 (10386) | (2) |
| *F. nucleatum rnfA::*Tn5 | Derivative of ATCC 23726 with Tn5 insertion into *rnfA* at position 511 (585) | (1) |
| *F. nucleatum rnfB::*Tn5 | Derivative of ATCC 23726 with Tn5 insertion into *rnfB* at position 222 (1158) | (2) |
| *F. nucleatum rnfC::*Tn5 | Derivative of ATCC 23726 with Tn5 insertion into *rnfC* at position 222 (1326) | (2) |
| *F. nucleatum* Δ*rnfC* | Isogenic derivative of CW1 lacking *rnfC* | This study |
| *F. nucleatum* Δ*rnfD* | Isogenic derivative of CW1 lacking *rnfD* | This study |
| *F. nucleatum* Δ*megL* | Isogenic derivative of CW1 lacking *megL* | This study |
| *F. nucleatum* Δ*radD* | isogenic derivative of CW1 lacking *radD*) | (1) |
| *F. nucleatum* Δ*kamA* | Isogenic derivative of CW1 lacking *kamA* | (2) |
| *A. oris* MG1 | Type strain | (3) |
| *S. oralis* 34 | RPS positive | (4) |
| *S. gordonii* DL1 | Type strain | (5) |
| *Plasmid* | | |
| pCWU6 | Derivative of pHS30 | (1) |
| pCM-GalK | *C. perfringens* vector expressing *galK* | (1) |
| pMCSG7-RnfC | Recombinant vector expressing His-tagged RnfC | This study |
| pRnfC | Derivative of pCWU6 expressing *rnfC* under the control of a *rpsJ* promoter | This study |
| pRnfD | Derivative of on pCWU6 expressing *rnfD* under the control of a *rpsJ* promoter | This study |
| pGalK--Δ*rnfC* | pCM-galK derivative; *rnfC* deletion vector | This study |
| pGalK--Δ*rnfD* | pCM-galK derivative; *rnfD* deletion vector | This study |
| pGalK--Δ*radD* | pCM-galK derivative; *radD* deletion vector | (2) |
| pGalK--Δ*megL* | pCM-galK derivative; *megL* deletion vector | (6) |
| pGalK--Δ*kamA* | pCM-galK derivative; *kamA* deletion vector | (2) |

**Table S2: Primers used in this study**

| Primer | Sequence[(a)] | Used for |
|---|---|---|
| rnfC-up-F | GGCGGGATCCATGAACTTTGAAGAAATAGATTTTTATATT | pGalK-Δ*rnfC* |
| rnfC-up-R | GGCGGGATCCTTAAAGGAGCTCCTATATGTTGTAAAAG | pGalK-Δ*rnfC* |
| rnfC-dn-F | GGCGGGTACCGTCCTATGGGGCTTGCACCACTTATG | pGalK-Δ*rnfC* |
| rnfC-dn-R | GGCGAAGCTTGCTAGTTGCTTCTGGTAAAACTTCTTTT | pGalK-Δ*rnfC* |
| com-rnfC-F | GGCGGGTACCGGATAGTAGAAGTGCATTTAAAGATT | pRnfC |
| com-rnfC-R | GGCGGGATCCCTACTTTTTCTTAGCTCTTAATTTAG | pRnfC |
| LIC-RnfC-F | TACTTCCAATCCAATGCAATGAAAGGAGTGTTT | pMCSG7-RnfC |
| LIC-RnfC-R | TTATCCACTTCCAATGTTACTACTTTTTCTTAGCTC | pMCSG7-RnfC |
| rnfD-up-F | CGCGGATCCAAAGGTATTGTTGGTATAGGAG | pGalK-Δ*rnfD* |
| rnfD-up-R | CCCATCCACTAAACTTAAACAATATGAGGAGCTGGTCCTGT | pGalK-Δ*rnfD* |
| rnfD-dn-F | TGTTTAAGTTTAGTGGATGGGTTTGCATTGGGATTAGGAGTTT | pGalK-Δ*rnfD* |
| rnfD-dn-R | CGCGTCGACAAATAATCCTAATACCTTATATAAG | pGalK-Δ*rnfD* |
| com-rnfD-F | GGGAATTCCATATGGTTTAGGAAATCCGGGCAAA | pRnfD |
| com-rnfD-R | CCGCTCGAGAGCTGCTATTAGACCAAGGA | pRnfD |
| radD-up-F | AAAGTCGACATGGTTTAGTGAAAGATTATTCAAAAT | pGalK-Δ*radD* |
| radD-up-R | AAAGGTACCATTTGCTCCA AAATCTATTT TATCA | pGalK-Δ*radD* |
| radD-dn-F | AAAGGTACCTCATCATCACCAATATTTAAGTCATTAG | pGalK-Δ*radD* |
| radD-dn-R | AAAGAGCTCCATAAATATCCTCAAAATATGAGTG | pGalK-Δ*radD* |
| kamA-up-F | GGCGTGAGCTCCAGAGATAGAAGTTTTTGATAAGGGTA | pGalK-Δ*kamA* |
| kamA-up-R | GGCGAGGTACCGTTTACCTTTCTACTACCATACCATAAT | pGalK-Δ*kamA* |
| kamA-dn-F | GGCGAGGTACCGGTACCAAAATAAAAAATGTTAGATAC | pGalK-Δ*kamA* |
| kamA-dn-R | GGCGAGTCGACAAGTAGCAATTTTTTCATTATTAGGAT | pGalK-Δ*kamA* |
| megL-up-F | CGCGGATCCGACATTCTCTTGAATTATAAAAAAAATCTG | pGalK-Δ*megL* |
| megL-up-R | AAAACTGCAGCCATTATAGATTTCTTTCCCATAACC | pGalK-Δ*megL* |
| megL-dn-F | TAACTTTACTCATTTGTCTTAATTCCTTAC | pGalK-Δ*megL* |
| RT-megL-F | CACAAGACTAGGCAATCCTACA | RT-PCR *megL* |
| RT-megL-R | GCTCCCATACCAGATGACATAG | RT-PCR *megL* |
| RT-cysK1-F | AACAGGGACAGGAGGTAGTT | RT-PCR *cysK1* |
| RT-cysK1-R | AGATGAAGCAGGCTCAACAG | RT-PCR *cysK1* |
| RT-cysK2-F | GCTACAAGTGGAAACACAGGA | RT-PCR *cysK2* |
| RT-cysK2-R | TCACTCATCCAATCTGGCATATAA | RT-PCR *cysK2* |
| RT-kamA-F | TCTCAATGGCAACTGGATTCTC | RT-PCR *kamA* |
| RT-kamA-R | TGCAGCATGGTCAACTGTATAA | RT-PCR *kamA* |
| RT-kamD-F | GTGCTGATGTTGTTGCAGTTAT | RT-PCR *kamD* |
| RT-kamD-R | TTCTTGTGTTGCCATTGTTCC | RT-PCR *kamD* |
| RT-radD-F | GCAGCAGCACCAACAATAAAT | RT-PCR *radD* |
| RT-radD-R | GGTGCTTCAGGAGGTGTTATC | RT-PCR *radD* |
| RT-16s-F | GGTTAAGTCCCGCAACGA | RT-PCR *16s* |

| | | |
|---|---|---|
| RT-16s-R | CATCCCCACCTTCCTCCTAC | RT-PCR *16s* |

[a] Underlined are restriction site sequences.

## References


1. Wu C, Al Mamun AAM, Luong TT, Hu B, Gu J, Lee JH, D'Amore M, Das A, Ton-That H. 2018. Forward Genetic Dissection of Biofilm Development by *Fusobacterium nucleatum*: Novel Functions of Cell Division Proteins FtsX and EnvC. mBio 9.

2. Wu C, Chen YW, Scheible M, Chang C, Wittchen M, Lee JH, Luong TT, Tiner BL, Tauch A, Das A, Ton-That H. 2021. Genetic and molecular determinants of polymicrobial interactions in *Fusobacterium nucleatum*. Proc Natl Acad Sci U S A 118.

3. Wu C, Mishra A, Yang J, Cisar JO, Das A, Ton-That H. 2011. Dual function of a tip fimbrillin of *Actinomyces* in fimbrial assembly and receptor binding. J Bacteriol 193:3197-206.

4. Wu C, Huang IH, Chang C, Reardon-Robinson ME, Das A, Ton-That H. 2014. Lethality of sortase depletion in *Actinomyces oris* caused by excessive membrane accumulation of a surface glycoprotein. Mol Microbiol 94:1227-41.

5. Hsu SD, Cisar JO, Sandberg AL, Kilian M. 1994. Adhesive properties of viridans streptococcal species. Microb Ecol Health Dis 7:125-37.

6. Chen YW, Camacho MI, Chen Y, Bhat AH, Chang C, Peluso EA, Wu C, Das A, Ton-That H. 2022. Genetic Determinants of Hydrogen Sulfide Biosynthesis in *Fusobacterium nucleatum* Are Required for Bacterial Fitness, Antibiotic Sensitivity, and Virulence. mBio 13:e0193622.

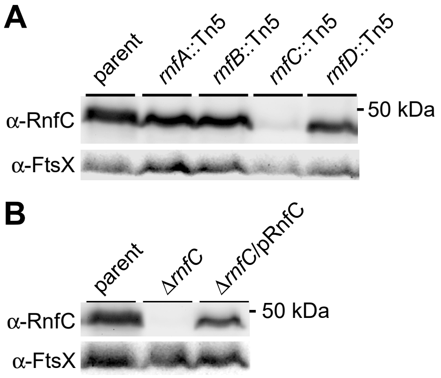


Figure S1: Britton et al.

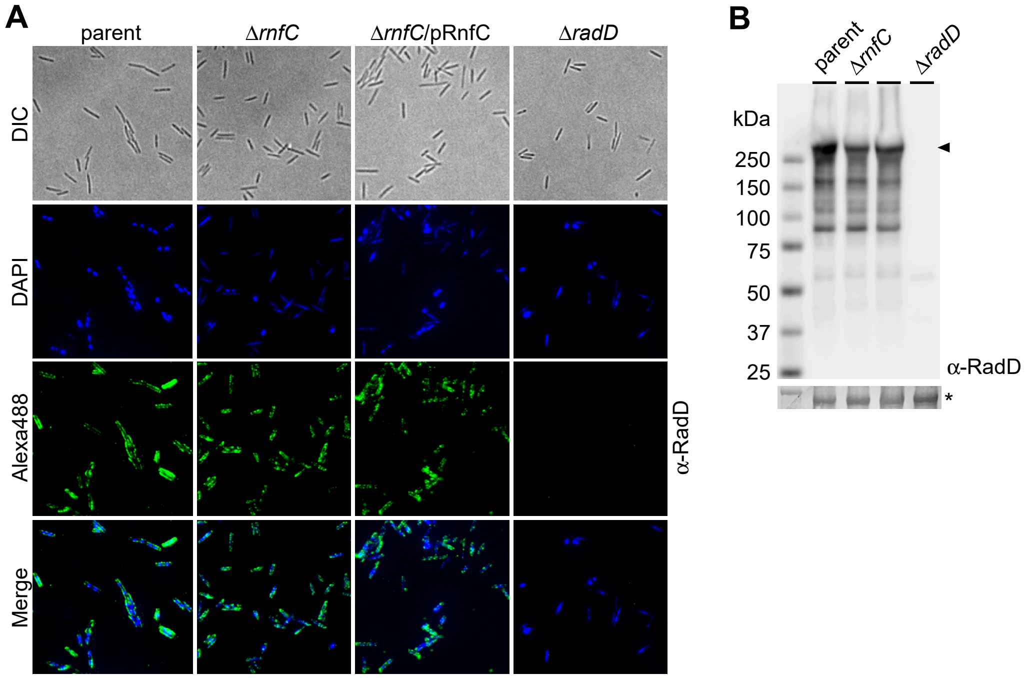


Figure S2: Britton et al.

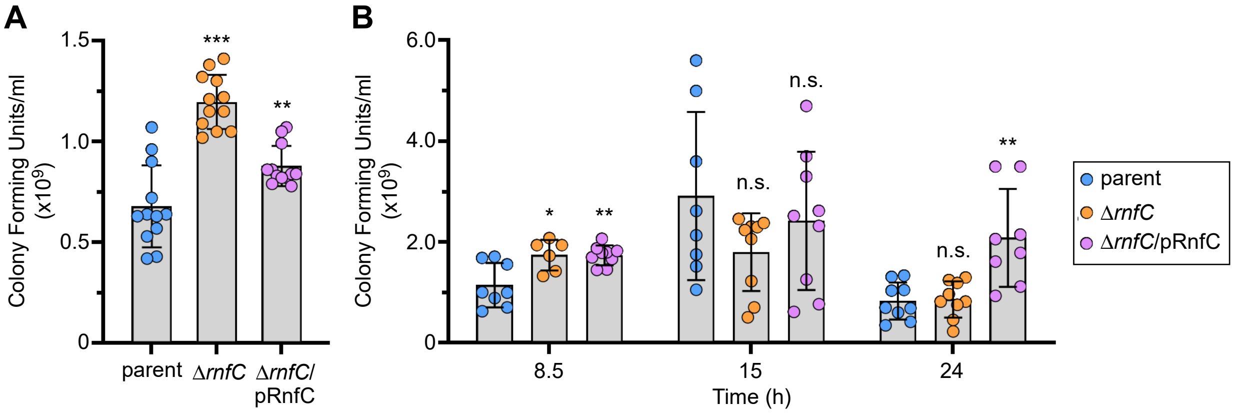


Figure S3: Britton et al.

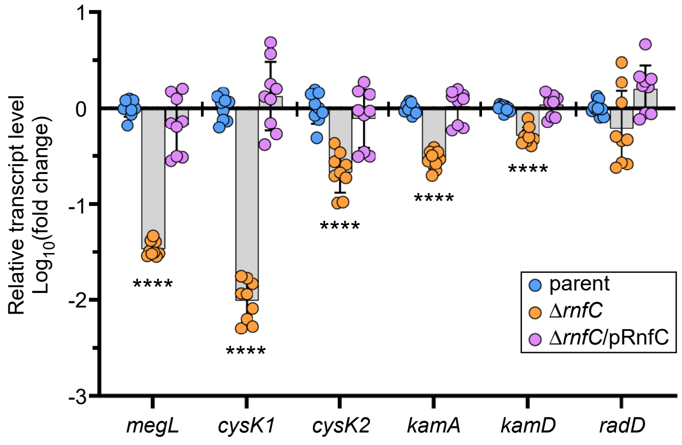


Figure S4: Britton et al.

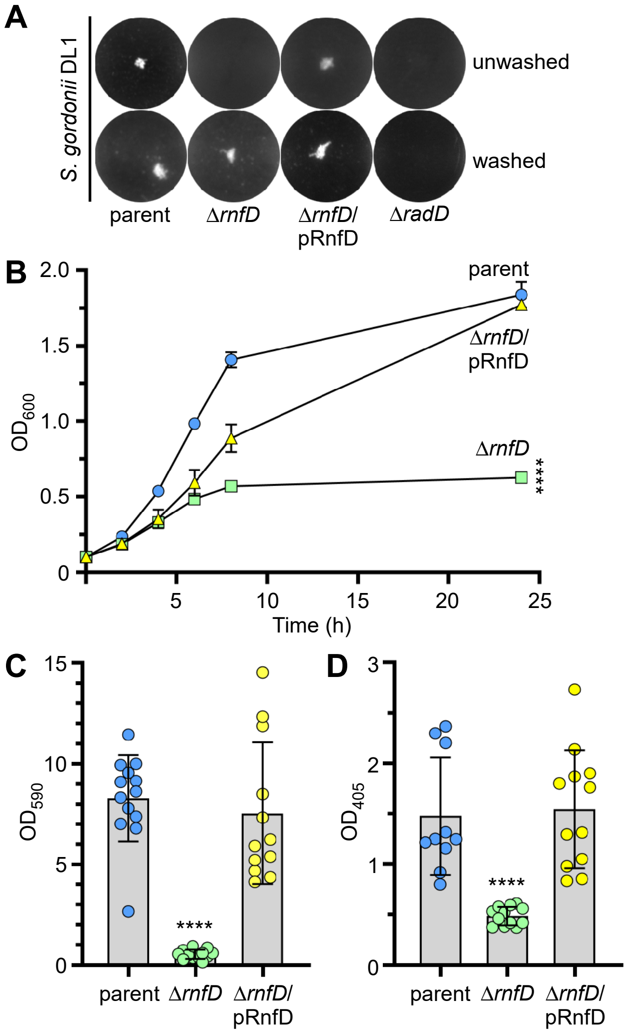


Figure S5: Britton et al.

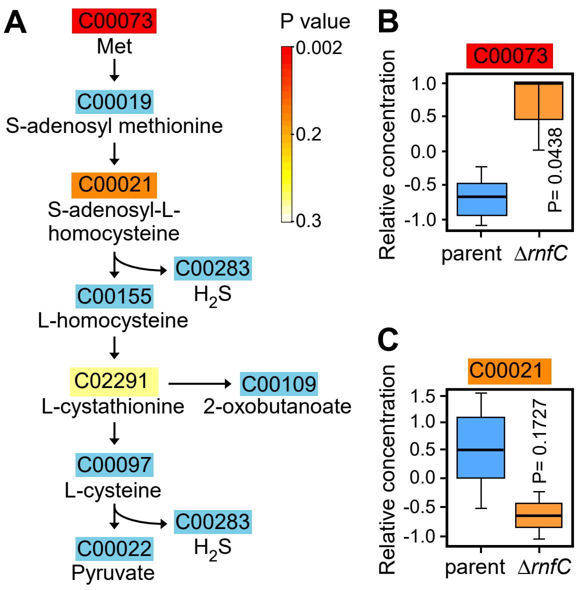


Figure S6: Britton et al.
